# Educational Possibilities of Keeping Goats in Elementary Schools in Japan

**DOI:** 10.3389/fvets.2016.00118

**Published:** 2016-12-26

**Authors:** Naoko Koda, Shiho Kutsumi, Toshiya Hirose, Gen Watanabe

**Affiliations:** ^1^Tokyo University of Agriculture and Technology, Fuchu, Tokyo, Japan; ^2^Nara University of Education, Nara, Nara, Japan

**Keywords:** children, education, elementary school, goat, interview survey, development

## Abstract

Many Japanese elementary schools keep small animals for educational purposes, and the effects and challenges have been investigated. Although goats are medium-sized animals that are familiar to Japanese, few practical studies have been conducted on keeping goats in schools. This study investigated the effects and challenges of keeping goats in elementary schools and discussed its educational possibilities. A semi-structured interview survey was conducted with 11 personnel that were responsible for keeping goats in 6 elementary schools in urban areas. They described benefits, problems, and tips related to keeping goats. Participant observation was also conducted on daily human–goat interactions in these schools. The results indicated that children in all six grades were able to care for goats. Goats were used for various school subjects and activities. As a result of keeping goats, children developed affection for them, attitude of respect for living things, greater sense of responsibility, and enhanced interpersonal interactional skills. Stronger ties between the schools and parents and community were developed through cooperation in goat-keeping. Some anxieties existed about the risk of injury to children when interacting with goats. Other challenges included the burden of taking care of the goats on holidays and insufficient knowledge about treatment in case of their illness or injury. The results suggested similarities to the benefits and challenges associated with keeping small animals in elementary schools, although the responsibility and the burden on the schools were greater for keeping goats than small animals because of their larger size and the need for children to consider the goats’ inner state and to cooperate with others when providing care. At the same time, goats greatly stimulated interest, cooperation, and empathy in children. Goats can expand educational opportunities and bring about many positive effects on child development.

## Introduction

To enrich the life experiences of children in Japan, elementary schools try to provide a variety of stimuli for their pupils such as an association with nature, as stipulated in the Education’s Guidelines for the Course of Study from the Ministry of Education, Culture, Sports, Science and Technology (1). Japanese elementary schools have a tradition of keeping animals for educational purposes, and animals are emphasized in environmental and science education (2). Today, most Japanese elementary schools keep animals (3), typically small animals such as rabbits, hamsters, birds, fish, and insects (4). Teachers tend to select these animals because they are discussed in textbooks and handbooks.

Various effects of keeping animals in elementary schools have been identified: nurturing children’s respect for life, sense of responsibility, affection, compassion, and friendship; healing hurt feelings; imparting knowledge about creatures; and so on (3–7). Animal-keeping promotes children’s psychosocial development and learning about subject such as living environment studies and science. However, proper management of animals is an important prerequisite, according to the 2001 declaration of the International Association of Human-Animal Organizations. Difficult aspects of animal-keeping in elementary schools include teachers’ insufficient knowledge about how to keep animals, the burden of animal care on holidays, treatment for illness and injury, care at the time of death, and so on (3, 4, 6). For these reasons, handbooks for teachers have been published [e.g., Ref. (8)], and veterinary medical association supports keeping school animals by providing teacher training and professional care in some areas (9).

The concerns above pertain to small animals. Medium-sized animals are more similar to children in body size than small animals, and they tend to interact with children more equitably. Medium-sized animals are recognized as useful educational resources when children visit zoos and observe them (10), but these effects are temporary.

The present study focused on the use of goats (*Capra hircus*) as medium-sized animals in elementary schools. Goats are livestock that are easy to keep (11) and have long been familiar to humans (12, 13), but few practical studies have been conducted on keeping goats in schools. Although, keeping goats has become rare in urban areas, this is gradually being reevaluated (13, 14). Koda et al. (15) showed that the experience of taking care of goats in elementary school raised awareness of symbiosis with goats among fourth-graders (9 or 10 years of age). This suggests goats can be useful in curriculum that develops scientific interest. To accomplish this, it is necessary to provide teachers with information that will help them take responsibility for the management and use of the goats.

This study investigated various factors involved in keeping goats in elementary schools and compared the results with those of previous studies about keeping small animals. This report summarizes the benefits and challenges of keeping goats that were recognized by the school personnel and discusses the educational possibilities of using goats. The process and changes in one school that replaced individual goats are reported, and recommendations for keeping goats in elementary schools are presented.

## Materials and Methods

### Participants and Schools

The principals of six elementary schools in urban residential areas in Japan in which goats were being kept were asked permission to conduct this survey, and all accepted. In each school, teachers were in charge of keeping goats, and two schools used other staff members to assist the teachers. A total of nine teachers and two staff members identified as potential main informants were invited to participate in this study, and all accepted.

Table 1 presents an overview of the schools. Grades of the children who took care of the goats differed among the schools, but across all six schools, children in all six grades of the Japanese elementary school system (6–12 years of age) participated in the care of goats. The children in the fifth and sixth grades who took care of the goats were members of animal care committees. At the start of the investigation, three schools were keeping goats for the first time. The other three schools were continuing to keep goats, and the participant teachers had taken over the workload from their predecessors. Among the experienced schools, one staff member participated who had been responsible for caring for the goats since the school started keeping them. The teachers were either classroom teachers whose students took care of the goats or advisers of the animal care committees. They were responsible for the practice of keeping goats and knew well the animals’ situation and the children who were involved in the practice. About 20–40 children were in each class with 2–4 classes in each grade that was standard in the areas. Five were public schools, and one was private. The goats were Japanese native breeds (*Shiba yagi* and *Tokara yagi*), which are easy to handle because of their small size (20–35 kg for adults) and tameness. They were health-inspected and treated with ivermectin by veterinarians before joining the program. Goats’ heath were checked regularly by veterinarians during the program also.

**Table 1:** **Summary of elementary schools surveyed**.

School	Caretaker grade	Caretaker type	Number of interviewees	Interviewee’s experience in keeping goats	Number of goats kept during survey period
A	1, 2, 5, 6	Grade (grade 1, 2), committee (grade 5, 6)	1	Novice	Two adults → one adult (one died), two babies (born)
B	2	Class	1	Experienced	One adult, two babies (born)
C	3 → 5, 6	Grade → committee	2	Novice	One adult → one adult (replaced), two juveniles
D	4	Grade	2	Novice	One adult
E	4	Grade	2	Experienced	One adult, two juveniles
F	5, 6	Committee	3	Novice, experienced	Two adults

### Data Collection

A semi-structured interview survey was conducted with the teachers and staff members in their schools. Survey items were divided into three parts according to the time that goats had been kept in the schools: past (procedures until goat-keeping began, sources of information about how to keep goats, and concerns before keeping goats), present (current situation of keeping goats, school topics and activities using or referring to goats, changes in children’s attitude and behavior, tips about keeping goats, and problems of keeping goats to be solved), and future and other prospects (plans for using goats in education, appropriateness of the current grade levels to take care of goats and explanations for this, recommendations to other grades or elementary schools about keeping goats, and advice they wished to provide). Each interview was about 30–60 min in duration and was conducted at a time that depended on the participant’s work schedule. The interview was conducted at several different times if necessary. All interviews were audio-recorded so that the content of the transcript could be analyzed. In addition, participant observations of daily human–goat interactions in these schools were conducted as a supplement to the survey.

## Results

### Past

The teachers and staff who were responsible for keeping goats talked about the procedure that had been in place until their schools began keeping goats. All schools had experience in keeping small animals, such as rabbits, chickens, hamsters, and fish. They expected that the effects of keeping goats would be providing children the chance to foster relations with nature and create bonds with creatures, to think about what they can do, to increase interactions with friends and society, to develop their social skills, to enhance their self-esteem and self-efficacy, and to calm their emotions. All schools were provided goats by farms or universities with agricultural department. Thus, the schools were able to receive professional advice and support in caring for the goats and could return them when they could no longer continue to keep them. The duration of goat-keeping experience in the schools varied from 1 year to nearly 30 years.

The sources of information about how to keep and use goats were their predecessors and teachers who had experience of keeping goats in other schools, farms, universities, books, websites, zoos, etc. All participants had in some way achieved some degree of knowledge about goats in advance. However, they were uneasy beforehand about care of the goats and interactions with them, including the burden of daily care, especially during holidays (two schools), insufficient knowledge about daily care and veterinary treatment (three schools), the possibility of injury, fear, and allergies among the children in their interactions with the goats (four schools), risk of failure to form bonds between children and goats (one school), and vague anxiety due to lack of general knowledge about goats (four schools). After initiating care of the goats, they noticed a gap between their knowledge and actual practice, and they tried to solve the problems through study, class discussion, and advice from experts and teachers with experience in keeping goats.

### Present

All of the schools had arranged a care system in accordance with their situations. On school days, the children divided into several groups and took care of the goats, taking turns before and after school and during break times between classes, and the teachers helped and supervised them. The children as a group cooperatively performed tasks such as cleaning the pen and paddock, preparing food, feeding, performing health checks, brushing, walking, and keeping written records. Children with allergies performed their tasks without direct contact with goats, for example, preparing food and recording. On holidays, other teams were formed of teachers, volunteer parents and children, and volunteers in the community, who took turns providing care. One school that was unable to devise a care system during a long vacation left the goats in the university that provided them. The pens and paddocks of the goats were fenced, but approach was not restricted, so that children who were not on care duty could watch the goats and interact with them during their free time, such as between classes.

The goats were used in various school subjects, for example, living environment studies (interaction, observation, sketching, etc.) in the first and second grades (three schools), science (body mechanism of animals and humans, environment, etc.) in the third grade or later (one school), Japanese (writing, diary-keeping, etc.; one school), integrated study (interdisciplinary classes; four schools), and moral education (bioethics, animal welfare, friendship, trust, etc.; two schools). Elements of various school subjects were involved in integrated study, and the children learned without noticing that they were studying. For example, they calculated the budget for goods necessary for their goat and measured the goat’s body parts (arithmetic), they recorded temperature and reviewed documents on goat birth (science), and they recorded daily care and gave reports (Japanese). The goats were also used as a teaching tool in various educational activities, including being involved in the annual curriculum (four schools); being introduced into classes, school events (e.g., excursions), and school assemblies (four schools); and being used in classes in other grades (two schools). The teachers recognized that the goats were a familiar hands-on tool for the children that stimulated their interest and understanding.

The participants in all schools recognized positive changes in children through keeping goats. Many psychosocial effects were identified. Participants in all schools said that the children developed affection for the goats and a sense of responsibility through caring for them. They also pointed out that the children actively learned about and observed the goats (four schools), learned the importance of life and came to respect the lives of other creatures and interacted with them affectionately (five schools), came to understand the minds of the goats and tried to adjust their behaviors toward them because they did not respond to verbal cues (five schools), were concerned about the goats, and developed compassion and cooperation with their friends (four schools). The goats also facilitated conversation and strengthened unity within the classes and groups (three schools). Unity within the children’s families and between school and families was also strengthened by children’s conversations about the goats at home (one school). In the school that kept multiple goats, the children noticed different personalities in each goat through close observation, and they came to respect individuality. The children willingly took care of the goats, and they developed autonomy and initiative in their daily lives (three schools). The children learned to tolerate to excrement and dirt, and they actively began to help clean at home (one school).

The goats sometimes had therapeutic effects on the children. The frequency of problem behaviors in school decreased (one school), children who were reluctant to go to school became more positive and came to enjoy school (four schools), and the goats soothed hurt feelings when unpleasant things happened at school (two schools).

It appeared that the goats would have an impact on children’s later lives. During the interviews, the participants mentioned the following possible long-term effects of goats. The children became more interested in creatures in general and in natural phenomena, and tried to understand them (four schools). The children showed interest in work related to animals and began to think about their future careers (one school). The children who took care of the goats also received higher overall evaluations in later grades (one school).

As for tips about keeping goats, all schools mentioned the importance of developing a system to cooperate and collaborate with parents, neighbors, and other personnel in schools so that they would understand goat-keeping as an educational activity and develop closer relationships. This contributed not only to the preventing problems in the neighborhood, such as noise and odor from the goats, but also to gaining voluntary support, for example, in caring for goats on holidays, maintaining facilities, providing information and instruction, contributing to the goats’ food supply (waste vegetables from homes, food stores, and school lunches and produce from the school garden), and expanding children’s interest and encouraging their learning with goats as common topics of conversation. At five schools, the participants recognized that their goats were outstanding features of the schools among members of the community. In keeping goats, the children had to make many decisions, such as negotiating their roles in the group and finding solutions when problems occurred. The participants explained that the teachers encouraged and supported the children to voluntarily notice the problems, examine and discuss the issues, and reach agreement and put it into practice, instead of the teachers making decisions and issuing commands (five schools). The teachers not only kept goats but also promoted their ripple effects in various educational activities. For example, a biotope and handmade nests for wild birds were located beside the paddock, and compost from goat excrement was used for produce in school garden.

On the other hand, participants also pointed out problems to be solved in keeping goats. These included the burden of maintaining the care system on holidays (two schools), concerns about coping with unusual bad weather (one school), constant worry about the animals (one school), the absence of livestock veterinarians in the neighborhood (one school), the risk of injury to children because of the goats’ large size (one school), and some children’s fear of the goats in the beginning (one school).

### Future and Other Prospects

The participants were asked about the prospects for goat-keeping in the future. Regarding new educational opportunities, teachers who had pregnant goats expected changes in children in response to the birth of the babies, and they also expected to give the offspring to other schools and thus create connections with them (two schools). Other plans and desires included using the goats in grades and classes other than the caregiving grade (one school), usage in environmental education and career education (one school), and deepening exchanges with the university that provided the goats (one school). Some participants recognized various constraints but wanted to take the goats to a large area such as the school grounds in order to facilitate interactions between children and goats (two schools) and to use the goats for weeding or landscaping (one school). Two schools wanted to continue in their present situation for a while and did not offer new plans.

When asked about the appropriateness of the current grades to take care of goats, participants of five out of six schools said this was satisfactory. The reasons were that the practice of keeping goats went relatively well and followed the curriculum for the grades. Keeping animals was appropriate for living environment studies in the first and second grades, and the study of animal and human body mechanisms was appropriate for science in the fourth grade (two schools). The participants whose lower-grade-children took care of goats mentioned the advantages that children in this age range, who are protected both in school and at home, became aware that they should protect others, such as their goats, and that many things do not go as well as expected. This experience led them to behave considerately and nurtured their ability to communicate feelings with others. Participants said that there is not enough room in the upper-grade curriculum to insert goats, and older children are busy with school events, committee activities, and subject studies. On the other hand, participants whose upper-grade-children took care of goats stated that children in the lower grades are too small to carry heavy feeders and experience safety risks, whereas children in the upper grades can do more physical work, and in terms of their psychosocial development stage, they can be more active in providing care and can cooperate with more responsibility. However, the participants in all schools admitted that goat-keeping is advantageous and possible in any grade, although the type of work required should be selected according to children’s developmental stage and curriculum. For example, if there is not enough room in the curriculum in the upper grades, goat-keeping can be offered as a committee or volunteer activity. Although the teachers of one school believed their goat to be unsuitable for the children in the current grade because of its large size, they thought that a smaller goat would be appropriate.

The final question was whether participants would recommend other grades or elementary schools that had not kept goats to begin this practice, and if they did recommend this, to state their reasons and provide advice. The participants in all schools said that they could not easily recommend this. Although they acknowledged many educational benefits of keeping goats, they recommended the practice only if an appropriate care system could be instituted, because the burden and responsibility were extensive and intense. The requirements were sufficient space for goats, a source of food and a water supply, and understanding and cooperation from the surrounding people, such as other personnel within the school, parents, neighbors, and experts (veterinarians or farmers with knowledge about goats). They stressed that once these requirements were satisfied, goats have many educational benefits as unique living teaching materials that are familiar to children, and their positive impact will compensate for the greater effort required of teachers with respect to other educational activities. Although some children may want to play during break times between classes instead of caring for the goats, the advantage of keeping goats is great, for example, children need to learn that not everything goes well according to their own will; children’s reactions to goat-related activities are clearly different from their reactions to other activities, such as arts and crafts; children can learn things they cannot learn at home; and children learn many things only from experience. Both goats and small animals have precious lives worth learning about, but the presence of goats is much more influential on children, and although burden of caring for goats is greater, their behavior is understandable, and they are tame livestock, resistant to disease and injury with a long life span.

### Improvement in Keeping Goats

All the schools prepared in advance and adjusted their methods of keeping goats by trial and error according to the situation and were able to use the goats in education. The participants recognized educational benefits and fulfillment while at the same time feeling some burden, and all wanted to recommend other grades or elementary schools to keep goats, if possible. In the interviews, only the participants of one school responded that their goat was unsuitable for the current caregiving grade. This school had an adult male goat for the first time, and because of its large size the school personnel felt it was a safety risk for the children in the current grade who were providing care. In addition, there were other teachers in the school who hoped to show children the birth of goats. After the initial survey, the school replaced the male goat with a pregnant goat. The male goat was given to a facility for adult people.

The effects of changing individual goats by this replacement were investigated in this school. Three years after the initial interview and participant observation, a similar survey was conducted. The informant in the interview was one of the teachers who had participated in the initial survey; he was asked to compare the current status with that of 3 years ago.

### Present (after Replacement)

The female goat gave birth twice in 3 years, and the school still had the goat and her two juveniles at the time of the second survey. An animal care committee was established in this school when the goat was replaced. On school days, the committee members in the fifth and sixth grades took care of the goats, taking turns performing the work before and after school, during break times between classes, and during cleaning time, and the teachers helped and supervised them. On holidays, other teams were formed with teachers and volunteer parents, and care was provided in the same ways as 3 years ago. At the time the goat was replaced, the school built a new hygienic pen, which was easy to clean and a fence around the paddock to ensure safety for children and goats. The teacher said that his concerns about the risks toward the children were considerably reduced, since the new goat was smaller and tamer. As with the previous male, for safety reasons, the teacher sometimes took care of the goat instead of the children. After replacing the goat, the children were able to undertake more work by themselves, and the teacher was able to encourage them to consider voluntary behaviors that would improve the quality of life of the goats. The teacher volunteered to be the adviser when the animal care committee was established. The teacher was able to take advantage of his past experience and knowledge but also learned in the course of interaction with the goats. As the result of births, there were five goats at one time. Due to restrictions of food supply and space and the burden of their care, the offspring were given to other facilities. The present goats’ vocalizations were sometimes different from those of the previous goat, and the teacher and children wondered what this meant. The teacher also felt regret that they gave up the male adult goat that the children had grown up with and felt affection toward, and he wondered if there had been something else they should have done for the goat instead of parting with it.

Regarding the changes in the children, the teacher reported his impression that the children came to feel that they took more care of the goats by themselves, compared to the former situation with the male goat, because the interactions between children and goats increased; for example, they came to be able to take the goats for a walk. The animal care committee was actually popular, and many children applied for membership, exceeding the committee capacity and requiring turnover among the members. Furthermore, although the official care was carried out by the animal care committee in the fifth and sixth grades, many other children, including those in the lower grades, came to visit the goats voluntarily and interacted with them freely in break times between classes. School subjects utilizing the goats were also expanded, such as sketching in drawing and manual arts, verbal expression in Japanese, and environmental education in integrated study.

### Future and Other Prospects (after Replacement)

As for the educational use of goats in the future, the teacher expressed the novel hope that the goats could participate with the children at athletic meets, although hygiene issues related to goat excrement on school grounds would have to be overcome in advance. Regarding the appropriateness of the current grades to take care of the goats, the teacher changed his judgment to appropriate because the children really enjoyed caregiving, and the size of the goats was appropriate for them. The teacher expressed his former opinion that he could not recommend that other graded or elementary schools that had not kept goats keep them unless the conditions of an adequate care system were satisfied, but he also wished to emphasize the benefits of keeping goats, if possible, because the positive impacts were great.

## Discussion

The results of this study clarified that goat-keeping is possible in all six grades of elementary school, despite the differences in children’s developmental stages and different circumstances of the schools. The teachers showed strong interest in the project, and in addition to providing daily caregiving tasks, the goats were used for teaching of different school subjects and activities according to children’s developmental stages.

### Effects of Keeping Goats

The teachers and school staff recognized the following effects of keeping goats: the children developed affection for the goats, attitudes of respect for living things, and a greater sense of responsibility, as a result of taking care of goats with others. They showed improvement in the quality of their school life and their interpersonal interactional skills, and their learning was promoted. Effects that were expected before the start of keeping goats were mostly achieved. These effects were consistent with previous reports on effects of keeping small animals in schools (3–7). Experience in interacting with animals offers children the opportunity to understand the similarities and differences between humans and animals and to learn non-verbal communication skills. Through a variety of real experiences, children who are physically and psychologically developing come to show interest in other creatures and non-living things, to understand relationships between the self and others and the environment, and to behave appropriately (16).

The advantages of keeping goats over keeping small animals included children’s development of greater compassion and sense of accomplishment, as well as greater happiness through interactions with an animal that they could not easily control with their own power. Because goats are medium-sized animals whose facial height is close to that of children, pupils could easily interact with them and observe their facial expressions and behavior. Goats have social cognitive abilities that make it possible for them to communicate with humans without specific training (17). Furthermore, children could not take care of the goats alone but needed to cooperate with friends, which led to strengthening of group cohesion. The children worked efficiently, assigning roles within the group. Since caregiving continues across the years, the children transferred their tasks by teaching the lower-graders how to take care of the goats. In the process, children were able to foster leadership skills and self-efficacy (18).

To achieve their common goal to improve the welfare of the goats, the children enhanced their understanding of others and cooperation and unity with them. These are important social skills, especially in Japanese society, which values group-oriented behaviors (19). Keeping goats should be a valuable experience for modern children, who are accustomed to playing video games that they can control by themselves as they wish (20).

Unlike small animals that are typically kept in classrooms, goats are medium sized, kept outside, and novel in the urban community. The goats attracted attention both inside and outside the schools. This may bring about connections among schools, children’s homes, and the community, developing stronger ties among them not only for keeping goats but also for other issues. This could lead to the formation of community schools. The schools in this study were located in urban areas, but there were senior neighbors who had lived on farms and kept goats or seen them daily when they were children. For them, goats were nostalgic animals. By contrast, goats are novel animals for present-day children. Regardless of age, people tended to show interest in the goats. Some schools became meeting places for children and neighbors, triggered by goat caregiving on holidays. Today, with urbanization and fear of crime and traffic accidents, children have fewer opportunities to play outside freely and interact with people of various ages (20–22). Goats can become a social lubricant that connects children with their parents and community, although there were people who felt the burden of caregiving on holidays.

Goats are different from pets because they are originally farm animals. They can also be living teaching material about systems of agriculture and the natural environment. Thus, in addition to the well-known effects of small animals, goat-keeping can be expected to have a strong impact on children’s learning about various fields, as well as a ripple effect in the community.

### Challenges of Keeping Goats

Common challenges between keeping goats and keeping small animals are the burden of caregiving on holidays as well as veterinary treatment in case of illness or injury (3, 4, 6). The differences are greater difficulty and responsibility in guaranteeing professional treatment in emergencies, in obtaining information about care, and in providing care to goats, which are unusual animals in urban areas. Generally speaking, the Japanese native breeds used in the schools in this study, *Shiba yagi* and *Tokara yagi*, are adapted to the environment in Japan and rarely need veterinary treatment. Moreover, unlike small animals (8), goats have a body size that reduces the risk that children will accidentally injure or kill them. All the schools in this study maintained cooperative relationships with farms or universities for professional treatment, but not all schools were located in areas that experts could immediately reach in case of necessity, unlike cases of health care for pets. In actuality, there were no reports in which experts were unable to arrive in time because of long distances, but the participants who were personnel responsible for goat-keeping were probably anxious in case of emergency.

Goats presented another risk different from small animals. There was the possibility that children would be injured by the goats’ horns when they shook their heads or by goat stepping on the children’s feet. Adults need to supervise when children are interacting with goats, and they must tell children to avoid accidents by always observing goats’ behavior and refraining from frightening them (23). The possibility of accidents can be reduced if children do not crouch beside the goats and do not make loud sounds or run, which can create panic in the goats because of fear. Keeping goats also requires more consideration toward neighbors, compared to keeping small animals. Vocalizations and odor of goats kept outside might be a nuisance. It is important to set pens and paddocks away from neighboring houses and to groom the goats and clean up the keeping sites regularly in order to reduce the odor. One way of reducing vocalizations is to choose male goats, because females frequently make high-pitched vocalizations while in estrus. But since vocalization is a natural behavior of goats, it is necessary to take this into enough considerations and ask neighbors’ understanding. In terms of zoonotic disease risks, all schools were instructed by veterinarians or farmers in advance to wash hands both before and after interaction with goats, in addition to veterinarians’ health check of the goats both before and during the program, and no case was reported about zoonosis.

The concerns that the participants expressed before they began keeping goats included the difficulty of maintaining a care system on holidays and the risk of injury to the children. However, it is possible to solve or reduce such problems. The schools in this study regarded the process of handling these issues as a positive part of education. A principal said, “If you encounter a problem, it is an opportunity. You can cope with problems one by one to come up with a solution, by thinking carefully. In the process, you can learn a lot.” The school that had safety concerns due to the large size of its goat was able to produce better effects across the grades by replacing the goat and changing its care system. In this process, they encountered new challenges, such as the need for greater space and food supply due to the birth of offspring, but they solved these problems. The schools turned issues that might have been barriers into educational opportunities.

### Future Possibilities

There were common effects and challenges between keeping goats and keeping small animals in elementary schools. However, the responsibility and burden were greater for keeping goats than for keeping small animals. This derives from the fact that goats are larger and children have to care for them while considering their inner states, and while cooperating with others. However, this is why goats stimulate interest, cooperation, and empathy in children, and bring about greater benefits. Benefits and challenges are two sides of the same coin.

The results of the present study suggest key factors in the popularity of keeping goats in elementary schools. When schools and teachers actively send out information about goats around them, people learn about the states and efforts of the schools and come to understand and cooperate with them. People are curious to visit the schools and share experiences with children, strengthening ties of interpersonal relations and increasing cooperation. Schools and teachers can obtain support from other organizations, such as local governments and veterinary medical association, for public and professional assistance and consultation in their community. Understanding and cooperation of parents and neighbors are also indispensable. Such factors make these schools distinctive in their community.

Goats can be kept by any grades of elementary school children in accordance with the purpose of their learning. The juvenile period is important in life span development in terms of cognition (24) and interpersonal relationships (25). Goats can play roles for children as teaching tools for cognitive development, as objects of empathy in social development, and as promoters of caregiving in non-cognitive development. Goats can be expected to expand educational opportunities and bring about many synergistic effects on child development.

The characteristics of goats as a species are worthy of consideration in animal selection as school animals. Goats are diurnal animals and thus consistent with the time of school activities. This is also advantageous in terms of animal welfare. Goats are herbivorous and can easily derive food from residues (11), such as weeds, fallen leaves, and leftovers of school lunches, making them almost self-sufficient. Their excrement contains little moisture, and it is easy to clean up and make into compost, which can be used for growing plants: children can learn thus material recycling. In addition, goats are of a moderate size to interact with children. Children sometimes treat small animals carelessly, and adults must pay more attention to their welfare (8). Although large animals increase the budget and risks to children, goats are relatively tame and easy in terms of health management. Also, in urban areas, the small number of animals and distance from other goats reduce the risk of infectious diseases, such as aftosa.

Each animal species has its own characteristics, and we should choose appropriate animals according to educational purposes. Compared with pets that can be kept at home (which are not necessarily kept at schools), large livestock that can be kept only on farms and wild animals that can be kept only in zoos (which children can learn about by visiting), goats are livestock that are difficult to keep at home but can be kept at schools. Goats are a meaningful choice for school animals, but it is necessary to establish a care system and accumulate useful knowledge and practices. The present survey results provide a guide to goat-keeping in schools to reduce anxiety and risk.

This study was a survey focused on participant observation and subjective responses by teachers and staff members in charge of goat-keeping. In the future, it would be useful to objectively examine the effects of keeping goats on children using more rigorous designs. Direct comparison of keeping goats with keeping small animals would also be meaningful, if comparable control groups can be established.

## Ethics Statement

Informed written consent was obtained from each participant before the study. The procedure was in accordance with the Code of Ethics and Conduct of the Japanese Psychological Association and was approved by each elementary school.

## Author Contributions

NK initiated the research, designed the study, and drafted the paper. SK performed the data analysis, wrote up the results, and revised the manuscript. TH proposed the idea and revised the manuscript. GW supervised the project and revised the manuscript.

## Conflict of Interest Statement

The authors declare that the research was conducted in the absence of any commercial or financial relationships that could be construed as a potential conflict of interest.
